# Recent Insights in the Paracrine Modulation of Cardiomyocyte Contractility by Cardiac Endothelial Cells

**DOI:** 10.1155/2014/923805

**Published:** 2014-03-13

**Authors:** Jacques Noireaud, Ramaroson Andriantsitohaina

**Affiliations:** Inserm UMR 1063 (Stress Oxydant et Pathologies Métaboliques (SOPAM)), Institut de Biologie en Santé-IRIS, 4 rue Larrey CHU, 49933 Angers Cedex 9, France

## Abstract

The cardiac endothelium is formed by a continuous monolayer of cells that line the cavity of the heart (endocardial endothelial cells (EECs)) and the luminal surface of the myocardial blood vessels (intramyocardial capillary endothelial cells (IMCEs)). EECs and IMCEs can exercise substantial control over the contractility of cardiomyocytes by releasing various factors such as nitric oxide (NO) *via* a constitutive endothelial NO-synthase (eNOS), endothelin-1, prostaglandins, angiotensin II, peptide growth factors, and neuregulin-1. The purpose of the present paper is actually to shortly review recent new information concerning cardiomyocytes as effectors of endothelium paracrine signaling, focusing particularly on contractile function. The modes of action and the regulatory paracrine role of the main mediators delivered by cardiac endothelial cells upon cardiac contractility identified in cardiomyocytes are complex and not fully described. Thus, careful evaluation of new therapeutic approaches is required targeting important physiological signaling pathways, some of which have been until recently considered as deleterious, like reactive oxygen species. Future works in the field of cardiac endothelial cells and cardiac function will help to better understand the implication of these mediators in cardiac physiopathology.

## 1. Introduction

The purpose of the present review is actually to shortly review recent new information concerning cardiomyocytes as effectors of endothelium paracrine signaling, focusing particularly on contractile function. For more information on the cardiac endothelial modulating factors and their roles in the regulation of other heart functions (growth, differentiation, rhythmicity, remodeling), please refer to some recent reviews [1–5].

**Figure 1:**
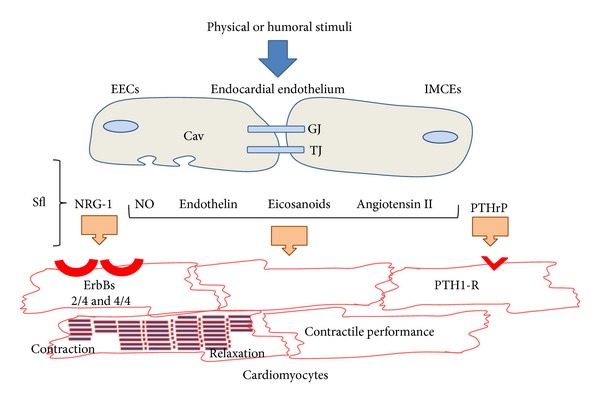
Paracrine communication between cardiac endothelial cells and cardiomyocytes. Endocardial endothelial cells (EECs) and intramyocardial capillary endothelial cells (IMCEs) respond to physical and humoral stimuli with the release of mediators such as nitric oxide (NO), endothelin 1, eicosanoid, and angiotensin II. These endothelial cells also release neuregulin-1 (NRG-1) acting on ErbB receptors on myocardial cardiomyocytes. IMCEs release parathyroid hormone-related peptide (PTHrP) which activates PTH1 receptor (PTH1-R) located on the cardiomyocytes. Gap junctions (GJ) allow cell-to-cell coupling for rapid intercellular communication of functional demands. Tight junctions (TJ, including zonula adherens) modulate the transendocardial endothelial-permeability through intercellular clefts. A large number of endothelial signaling molecules localize to caveolae (Cav). Sfl: subendocardial fibroelastic layer (or extracellular matrix including sympathetic nerve fascicles).

First of all, it is important to make the distinction between the respective contribution of the cardiac endothelial cells in the myocardial capillaries and at the endocardium (purpose of the present review) [6]. The cardiac endothelium is formed by a continuous monolayer of cells that line the cavity of the heart (endocardial endothelial cells (EECs)) and the luminal surface of the myocardial blood vessels (intramyocardial capillary endothelial cells (IMCEs)). EECs are the first of the endothelium cells to develop and originate from the cardiogenic plate by the process of vasculogenesis, whereas the IMCEs originate from the mesothelial cells of the epicardium, by angiogenesis. The luminal surface of the majority of EECs has a variety of microvilli that project into the heart cavities [7]. The large contact surface area of the endocardial endothelium with cardiac cells suggests an important sensor role for EECs [8]. Gap junctions, tight junctions, and zonula adherens are present between EECs where they play a role in rapid intercellular electrochemical coupling as well as to act as a selective barrier to limit the paracellular diffusion of molecules through the intercellular spaces, respectively [8, 9]. Golgi apparatus and endoplasmic reticulum of EECs are abundant with a great number of mitochondria surrounding the nucleus [9] suggesting that these cells are highly active metabolically. Similarities exist between EECs and IMCEs, but differences are also present between these two types of endothelial cells. An important feature of endothelium is also the presence of numerous caveolae. Caveolae are small (70–90 nm in diameter) specialized invaginations of the plasmalemmal membrane. These organelles are present in most mammalian tissues and are particularly abundant in endothelial cells. A large number of signaling molecules that regulate endothelial cells localize to caveolae (for recent review, see [10]). Caveolae are abundantly provided in caveolin-1 (Cav-1) which constitutes the nonmuscle isoform of a coat protein of caveolae. Brutsaert has reviewed in detail this point [1]. Thus, immunostaining for Cav-1 shows that the peripheral borders of EECs are nearly completely devoid of caveolin labeling, whereas IMCEs display a very intense labeling for Cav-1 [11]. Caveolin-rich plasmalemmal microdomains are sites for the constitutive nitric oxide (NO) synthase (eNOS), and the poverty of Cav-1 in these areas suggests that eNOS activity might be associated with membrane components other than caveolae or with parts of the cytoskeleton. IMCEs do not have gap junctions; thus these cells differ in the way they communicate with other adjacent endothelial and nonendothelial cells [8]. Considering their respective cytoskeleton components, stress fibers, vimentin filaments, and microtubules are found to be different in EECs and IMCEs. IMCEs contain more actin filaments or stress fibers compared to EECs. Vimentin filaments and microtubules are closely packed and aligned parallel to the cell axis in IMCEs, whereas in EECs, these components constitute an extensive filamentous network. EECs interact with components of the circulating blood entering and leaving the pulmonary vasculature and both act as an autocrine or paracrine system that modulates cardiomyocyte function and as a barrier between the superfusing blood and the cardiomyocytes. EECs and IMCEs play a role in controlling the contractility of cardiomyocytes by releasing various factors. In the normal adult heart, cardiac endothelial cells produce NO through the activity of eNOS, endothelin 1 (ET-1) after conversion of pre-proET-1 to proET-1 and into ET-1 by the ET converting enzyme, eicosanoids as prostaglandins, transform angiotensin I (Ang I) into active angiotensin II (Ang II) and release peptide growth factors and neuregulin-1 (Figure 1).

## 2. eNOS and NO

The eNOS is mainly present in both EECs and IMCEs [11], but to lesser extent also in cardiomyocytes [12]. Immunostaining experiments have shown that there is a considerable nonuniformity of eNOS expression between EECs and IMCEs. eNOS is generally associated with the particulate fraction in endothelial cells, in particular with the Golgi complex and with domains of the plasma membrane, the caveolae. The more intense eNOS staining in EECs compared to IMCEs appeared to be associated with more intensely labeled and larger Golgi complexes. Heterogeneity is characteristic also for eNOS labeling of the peripheral cell borders between EECs and IMCEs. The eNOS-stained peripheral borders are distinct in EECs and not observed in IMCEs. NO production by the peripherally located eNOS in EECs may be involved in the regulation of paracellular permeability and suggests a greater eNOS activity in these cells than in IMCEs. These two cell types probably account for most of the NO measurable in the effluent of* in vivo* heart or whole cardiac preparations.

Studies in endothelial cells reveals that eNOS targeted to the plasmalemma releases greater amounts of NO compared with Golgi tethered eNOS [13] and the increased amount of NO produced greatly influenced the mechanisms implicated in NO effects (cGMP-dependent signaling versus S-nitrosylation of target proteins with high and low concentrations of NO, respectively; see below) [14]. Considering eNOS associated with caveolae, eNOS interacts with Cav-1 that ensures the proper targeting of eNOS to caveolae and maintains eNOS in an inhibited state. This inhibition can be reversed by addition of exogenous calmodulin, suggesting a reciprocal regulation of the enzyme by Cav-1 versus activating calcium-calmodulin [15, 16]. Stimulus- or agonist-induced increases in intracellular calcium promote the dissociation of Cav-1 and eNOS complex. Binding of activated calcium-calmodulin to its consensus sequence on eNOS initiates catalytic activity. The phenotype of mice deficient in Cav-1 exhibits a marked hyporesponsiveness to constrictor agonists attributable to an increase of NO release [17, 18]. It has also been shown that statins potentiate eNOS activity by decreasing Cav-1 abundance* in vitro* and* in vivo*, at least in macrovascular endothelial cells where the caveolin pool is lower and the proportion of caveolin-bound eNOS is higher [19] (see also review in [4]). However, another protein called NOSIP (for eNOS-interacting protein) has been also described to be able to target eNOS to caveolae in endothelial cells of the cardiac microvasculature in rat heart [20]. These authors suggest that NOSIP is a novel type of modulator that promotes translocation of eNOS from the plasma membrane to intracellular sites, thereby uncoupling eNOS from plasma membrane caveolae and inhibiting NO synthesis.

Experiments in cultured endothelial cells have demonstrated that eNOS expression can be modulated by many factors including shear stress, TGF-*β*, protein kinase C, TNF-*α*, oxygen, and the proliferative state. The ubiquitous 90 kDa, heat-shock protein (Hsp90), is expressed at high levels (accounting for up to 1-2% of total cellular protein content) in the cytosol even in unstressed conditions. Hsp90 functions as a chaperone for the proper folding of specific protein substrates include many signal transducing molecules (e.g., nonreceptor tyrosine kinases, transcription factors, and eNOS, among others; for a review, see [21]). Most of its regulatory action in eNOS signaling has been described in endothelial cells. Hsp90 is associated with eNOS in resting endothelial cells, and upon stimulation with vascular endothelial growth factor (VEGF), estrogen, histamine, shear stress, and statins, the association between the two proteins is increased, resulting in enhanced NO production [22]. The protein kinase Akt, the kinase involved in the phosphorylation of eNOS on the active site serine 1177, is another scaffold protein for Hsp90. Akt binds to a sequence of Hsp90 that does not overlap with that involved in the binding of eNOS. Therefore, Hsp90 is proposed as an adaptor between Akt and its substrate, eNOS, thereby promoting the phosphorylation of eNOS at the active site (for more details, see [23]).

The main physiological source of NO in normal, adult nonstressed cardiac tissue is eNOS from EECs and IMCEs. The effects of NO on myocardial contraction and relaxation have been very much studied and the signaling pathways in the heart have been reviewed in detail [4, 24, 25]. A positive inotropic response to low concentrations of NO has been reported in several studies in isolated cardiomyocytes [26, 27], whereas it induced a negative inotropic effect only at higher concentrations [28].

Most of the negative actions of high concentrations of NO on cardiac performance are due to the activation of myocardial soluble guanylate cyclase to produce cGMP and desensitization of cardiac contractile myofilaments to calcium [29]. The positive lusitropic effect of NO has also been attributed to cGMP- and PKG-mediated phosphorylations of troponin I, subsequent to myofilament calcium desensitization, relaxation hastening, and improved distensibility [30].

NO exerts its positive inotropic effects mainly via a mechanism independent of cGMP. Very low concentrations of NO activate G proteins such as Gs, stimulating adenylyl cyclase, and the L-type calcium current (*I*_CaL_) from cardiac voltage-dependent L-type calcium (VDLC) channels and Gi, hence stimulating muscarinic K^+^ channels [31]. NO may also bind superoxide anions to form peroxynitrite, which exerts specific effects on cardiac voltage-dependent channels and contraction [32–35]. It is indeed important to remember that NO can exert its effects directly through the S-nitrosylation of target proteins [36–40]. A recent proteomic study identified as many as 951 unique proteins that can be S-nitrosylated in the heart [41], demonstrating its potential importance in regulating cardiac function. Indeed, direct nitrosylation of the ryanodine receptor RyR2 accounts for the enhanced excitation-contraction coupling gain and positive inotropic effect of cardiomyocyte stretch [42]. A direct S-nitrosylation of the cardiac VDLC channels through a redox switch-mediated increase in *I*_CaL_ could also be involved [32, 33]. Although cGMP-independent activation of adenylyl cyclase at low NO levels has been suggested [26], a cGMP-dependent increase in cAMP, through cGMP-mediated inhibition of cAMP phosphodiesterase III (PDE3) and prevention of cAMP breakdown, has been reported [43]. PDE3 and PDE4 are actually considered as critical regulators of cAMP signals in cardiomyocytes [44] (for recent review on PDEs, see [45]).

## 3. Endothelin-1 (ET-1)

EECs and IMCEs are the major source of ET-1 in the normal heart [46], and cardiomyocytes are its primary target. Cardiac tissue displays a high density of ET_*A*_ and ET_*B*_ receptors in cardiomyocytes and endothelial cells [47, 48]. ET-1 is one of the most potent positive inotropic agents known [49, 50]. Its positive inotropic effect has been partly explained by an enhanced affinity of the contractile proteins to calcium [51], subsequent to an intracellular alkalosis resulting from activation of the sarcolemmal Na^+^/H^+^ exchanger [52]. Thus, ET-1 might reverse acidosis-induced negative inotropic and lusitropic effects, without increasing intracellular calcium as it occurs with most positive inotropic agents. Moreover, it has been shown that ET-1 binds directly* in vivo* to the ET_*B*_ receptors on the endothelial surface and induces the release of endothelium-derived relaxing factors, such as NO and prostanoids [53], rather than directly promoting myocardial inotropy through ET_*A*_ receptors on the cardiomyocytes (reviewed in [3, 5]).

Furthermore, NADPH oxidase-derived reactive oxygen species (ROS) play a physiological role in the acute regulation of cardiac contractility in the intact rat heart. Results of Kubin et al. [54] reveal that ET-1-induced increase in cardiac contractility is partially dependent on enhanced ROS generation, which in turn activates the extracellular signal regulated kinase 1/2 (ERK1/2)-p90 ribosomal S6 kinase-Na^+^/H^+^ exchanger-1 pathway [55]. It is known that excessive levels of ROS can modulate the activity of different proteins involved in the excitation-contraction coupling, including the sarcoplasmic reticulum (SR) Ca^2+^ release channel, the SR Ca^2+^ ATPase, and the L-type Ca^2+^ channel, by modifying sulfhydryl groups of cysteine residues [56, 57]. In contrast to pathological conditions, less is known about the role of ROS in the regulation of normal cardiac function. Some data support the existence of a relationship between contraction, oxidative metabolism, and ROS production in the cardiomyocytes. Thus, increases in contraction frequency are accompanied by enhanced oxygen consumption and ROS formation in isolated cardiomyocytes [58, 59]. However, the effect of ROS on contractile function is controversial and prior studies have produced conflicting results regarding the role of ROS and ET-1 in the regulation of contractile function in isolated cardiomyocytes. Indeed, Ang II induces ET-1 release and an increase in ROS generation, which in turn triggered an increase in contractility in cat cardiomyocytes [60]. Moreover, it has been suggested that the positive inotropic effect of exogenous ET-1 is almost exclusively dependent on ROS production in this model [61]. ET-1 has been shown to increase contractility in several species such as rat, rabbit, cat, guinea pig, and human [62, 63]. However, ET-1 can elicit both positive and negative inotropic effects in murine models. Thus, ET-1 induces a negative inotropic effect in isolated mouse cardiomyocytes [64].

## 4. Prostaglandins

Endothelial cells, including EECs and IMCEs, synthetize and release several prostaglandins in response to a wide variety of hormonal, chemical, immunological, and physical stimuli [65, 66]. Cyclooxygenases (COX) in both constitutive (COX-l) and inducible (COX-2) isoforms are key regulators of prostaglandin synthesis. COX-l is constitutively expressed in all endothelial cells in the heart and is believed to provide cytoprotective effects. COX-2 is nearly undetectable under normal physiological conditions but is induced in endothelial cells and in macrophages during inflammation.

Brutsaert [1] has reviewed in detail the differences between EECs and IMCEs on prostanoid productions. Mebazaa et al. [67] have reported abundant production of prostacyclin (PGI_2_) and prostaglandin (PG)E_2_ from cultured EECs from right and left bovine ventricles, with EEC production of PGI_2_ being 10 times higher than that of PGE_2_. On the contrary, IMCEs have been reported to release more PGE_2_ than PGI_2_. The reasons why IMCEs release more PGE_2_ than PGI_2_ are still unknown. Responses to PGI_2_ and PGE_2_ range from increased inotropy [68] to no effect [69], to negative inotropy [70].* In vitro* studies on isolated papillary muscle have helped to explain some of those inconsistencies [71]. With the use of activators and inhibitors of endogenous eicosanoids and of NO in different combinations, the inotropic action of eicosanoids and NO is reciprocal. Thus, stimulation of endogenous prostaglandin (mainly PGI_2_) release abolishes the inotropic effect of NO, in particular on the onset of relaxation, while demonstration of a PGI_2_-induced positive inotropic (contraction-prolonging) effect is dependent on inhibition of NO synthesis. Accordingly, PGI_2_ and NO interact to regulate cardiac contractile performance, by their opposing effects on the onset of myocardial relaxation. NO, PGI_2_, and, to a smaller extent, PGE_2_ share a number of important properties and that their synthesis and release from endothelial cells are often coupled through continuous cross talk between the NOS and COX pathways [72], although their underlying subcellular mechanisms await further clarification. Their mutual actions on the target cardiomyocytes are closely linked, largely through the effects of PGI_2_ and NO in establishing cAMP-to-cGMP ratios rather than absolute intracellular concentrations of cAMP or cGMP. The localization of PGI_2_ synthase at the intercalated discs favors a new function of PGI_2_ in the heart [73]. Since the PGI_2_-receptor (IP-receptor) is also present in the same compartment, cAMP-dependent phosphorylation of connexin 43 leading to increased electrical coupling of cardiomyocytes may play a physiological role of PGI_2_ in these cells.

In mouse atria, acetylcholine induces a biphasic inotropic response; that is, a transient decrease in contractile force is followed by a late increase mediated by muscarinic M2 and M3 receptors, respectively [74]. Tanaka et al. [75] show that the positive response is mediated by prostaglandin released from the endocardial endothelium. In ventricular myocardia, it has been reported that PGF_2_ induces a positive inotropic response and a rise in intracellular pH (i.e., an alkalosis) [76]. In the case of the mouse atria, Tanaka et al. [77] do not detect change in pH in response to PGF_2_. Further studies are needed to better understand the inotropic effect of PGF_2_.

## 5. Angiotensin II (Ang II)

Most of the effects of Ang II on cardiac contractile performance result from locally produced rather than from circulating Ang II [78]. Ang II is synthetized through both Ang convertase enzyme- (ACE-) dependent and ACE-independent pathways, expressed in EECs and IMCEs. Several studies have investigated whether Ang II affects cardiomyocyte contractility. As an acute response to Ang II, some authors find a negative contractile response in mouse cardiomyocytes [79]. Others report a direct positive inotropic response in isolated cardiac preparations [60, 80]. In human ventricular myocardium, Ang II has either no effect or it exerts positive inotropic responses [81, 82]. The effects of Ang II share some features of *α*-adrenoceptor stimulation. For example, both agonists are able to activate PLC-dependent pathways. However, in contrast to *α*-adrenoceptor stimulation, Ang II induces ROS formation through NADPH oxidase activation and stimulates stress-activated pathways as well [83, 84]. On the mechanistic basis, the different responses evoked by Ang II are mediated by activation of either G*α*q or G*α*12/13-coupled receptors. Ang II-dependent ROS formation, probably from NADPH oxidase, induces the expression of TGF-*β*_1_ in cardiomyocytes [83, 85, 86]. Mufti et al. [87] provide strong evidence that TGF-*β*_1_ is a key player mediating the Ang II-dependent long-term cardiodepressive effect. Ang II causes a negative contractile effect in adult rat cardiomyoctyes and this has been found to depend on p38 MAP kinase activation via ROS-independent formation [88]. Mufti et al. [87] also report that the effects of Ang II on cell shortening are also dependent on p38 MAP kinase pathway. However, as the effect in this study depends on Ang II-dependent TGF-*β*_1_ activation, one can expect the participation of ROS formation. The exact role of p38 MAP kinase requires further studies [89]. de Giusti et al. [90] recently reviewed that acute activation of the cardiac renin-Ang II-aldosterone system induces mitochondrial ATP-dependent K^+^ channel opening and subsequently enhances the production of mitochondrial ROS. These oxidant molecules, in turn, activate membrane transporters, as the Na^+^/H^+^ exchanger (NHE-1) and the Na^+^/HCO cotransporter (NBC)* via* the stimulation of the ROS-sensitive MAPK cascade. The stimulation of such effectors leads to an increase in cardiac contractility. The mechanism of how the activation of NHE-1 or NBC regulates cardiac contractility involves the increase in intracellular Na^+^ concentration [91]. Indeed, the activation of these transporters leads to a subsequent increase in intracellular Ca^2+^ concentration due to the activation of the reverse mode of the Na^+^/Ca^2+^ exchanger [92–95].

## 6. Peptide Growth Factors

Only preliminary data are presently available about a possible role for peptide growth factors in the performance of the adult heart. IMCEs express and release parathyroid hormone-related peptide (PTHrP) [96, 97]. PTHrP exerts a positive inotropic, chronotropic, and lusitropic effects in adult ventricular cardiomyocytes and PTHrP released during an ischemia improves the inotropy of the postischemic heart [98]. PTHrP via PTH1 receptor (PTH1-R) directly improves cardiac function and myocardial perfusion through protein kinase A/protein kinase C-dependent activation of adenylate cyclase [99]. Evidences have been provided that PTHrP may play a role regarding cardiac dysfunction during situation with NO deficiency such as menopause. Indeed, Schreckenberg et al. [100] report that chronic NO deficit is associated with a loss of the inotropic and chronotropic effect of this hormone resulting from downregulation of PTH1-R via TGF*β*_1_-dependent pathway in left ventricular cardiomyocytes.

## 7. Neuregulin-1 (NRG-1)

Neuregulin-1 (NRG-1), a growth factor released from cardiac endothelial cells, has been shown to be essential for the normal function of the adult heart (Parodi and Kuhn [101]). NRG-1 mediates its actions through activation of the extracellular domain of the tyrosine kinase receptors, ErbB. In the adult heart, NRG-1, ErB2, and ErB4 are found in cardiomyocytes. Binding of NRG-1 to ErB2 and/or ErB4 induces the formation of homo- and heterodimers on cardiomyocytes [102]. Although NRG-1 does not bind directly to ErB2, it is the favored coreceptor for heterodimerization [103]. Thus, in adult cardiomyocytes, NRG-1 signaling occurs through ErbB2/ErbB4 heterodimers and/or ErbB4/ErbB4 homodimers. The first evidence for a role of NRG-1/ErbB signaling in adult heart function comes from clinical studies in patients with metastatic mammary carcinoma, undergoing combination therapy. Trastuzumab (Herceptin), a monoclonal antibody against ErbB2 combined with an anthracycline, elicited dilated cardiomyopathy and heart failure [104]. Using targeted gene inactivation in the mouse, different studies support the hypothesis that NRG-1 plays an essential roles during development of the heart and peripheral nervous system and demonstrates that the main NRG-1 receptors* in vivo* correspond to ErbB2/ErbB4 or ErbB2/ErbB3 heteromers, respectively (for reviews, see [105, 106]). The receptors ErbB2 and ErbB4 remain expressed in adult cardiomyocytes. They are localized to the T-tubule system and intercalated discs in the vicinity of components of the excitation-contraction machinery [107–109]. Thus, both the *α*- and *β*-isoforms of NRG-1 induce a negative inotropic response and activate NOS in isolated adult rabbit papillary muscles [110]. These authors also show in neonatal rat cardiomyocytes that NRG-1 can activate Akt leading to the phosphorylation of eNOS [111]. Thus, the NRG-1/ErbB signaling pathway may have a modulatory role and could be activated in conditions of enhanced cardiac inotropism, such as in myocardial hypertrophy or during *β*-adrenergic overdrive. On the other hand, dynamic regulation of NRG-1 expression in EECs and IMCEs occurs* in vitro* in response to other endothelial factors. Thus, mechanical stretch or stimulation with ET-1 leads to upregulation of NRG-1, while prolonged treatment with Ang II results in downregulation of its expression [112].

## 8. Pathophysiological Role of Cardiac Endothelial Cells

As reviewed above, EECs and IMCEs are modulators of ventricular cardiomyocyte contractile function. Thus, damage and/or dysfunction of cardiac endothelium could have a serious impact on the development of cardiac diseases. A deficient production of NO by eNOS contributes to diastolic ventricular dysfunction and abnormalities in Ca^2+^ homeostasis. Alterations in NO generation or disruption in its targeting have been shown in various pathological conditions like atherosclerotic vascular disease, congestive heart failure (CHF), and essential hypertension (reviewed in [2, 113–115]). Indeed, eNOS can be a major ROS generator during pathologic stress. Dysfunction of NO pathway via eNOS participates in cardiac arrhythmia. Indeed, eNOS deficient mice display a slower heart rate and increase in the transient inward current and tachycardia [116]. Although cardiomyocytes isolated from heart of eNOS deficient mice have normal resting action potential (AP) duration and VDLC current under basal condition, their response to isoproterenol is altered. Application of isoproterenol induces longer AP duration, with increases in early and delayed after depolarization, and therefore induces cardiac dysfunction [117]. It is generally known that alteration of cardiac contraction is one of the main causes of cardiac remodeling leading cardiac hypertrophy. Evidences have been provided on the role of eNOS in these pathologies. Indeed, chronic eNOS deletion induces concentric hypertrophy and worsens remodeling after pressure overload [118, 119]. Other authors using abdominal aortic banding found increased hypertrophy but less chamber dilation in eNOS deficient mice [120, 121]. The therapeutic approaches used for improving NOS function in cardiovascular diseases have used organic nitrates to increase circulating NO level* via* denitration [122–124]. Enhancement of NOS pathway using various compounds has been recently described as therapeutic approaches for treating cardiac diseases and these include arginase inhibitors, NOS activators, NOS transcription enhancers, NOS recouplers, soluble guanylate cyclase activators, PDE5 inhibitors, and antioxidants (reviewed in [115]). ET-1 may also be involved in the modulation of contractile performance in pathological states. Enhancement of ET-1 production has been detected in various cardiovascular stress including acute myocardial infarction, ischaemia, CHF, cardiogenic shock, and oxidative stress (for reviews, see [2, 3, 5]). Thus, targeting ET-1 system may be therapeutic benefit for cardiovascular diseases. Indeed, bosentan and ambrisentan, ET_*A*_-ET_*B*_ receptor, and selective ET_*A*_ receptor antagonists, respectively, have been shown to be successful in treating right ventricular hypertrophy and right heart failure subsequent to pulmonary arterial hypertension [125, 126]. However, ET-1 receptor antagonists have not yet proven to be efficient for the treatment of CHF [5, 125].

## 9. Conclusions

The modes of action and the regulatory paracrine role of main mediators delivered by cardiac endothelial cells upon cardiac contractility identified in cardiomyocytes are exceedingly complex and are not fully described. Furthermore, as pointed out by and fully referenced in Brutsaert [1] “*a still higher scale of complexity in the in vivo intact heart may ensue from their interaction with other important cardiomodulatory pathways, such as the *β*-adrenergic or cholinergic pathways in the heart, atrial and brain natriuretic peptide activity, and circulating thyroid and aldosterone hormones.*” Moreover, differences in the contribution of transporters and pumps to Ca^2+^ homeostasis between rodents and humans should be taken into account. Thus, the challenge to translate experimental evidences obtained in single cardiomyocytes to the whole heart and into useful novel therapeutic strategies is still rather complicated. Careful evaluation of new-targeted therapeutic approaches is required not to alter important physiological signaling pathways. A relevant example illustrating such requirement is the heuristic shift that occurred in recent years, showing that ROS can act as intracellular signaling molecules playing important nonpathological roles in different physiological mechanisms (present review; see also [127]). Nevertheless, the present review summarizes some insights regarding the control of cardiac contractility by endothelial cells mediators. This information may help future works to fight against cardiac diseases.
